# Role of C-reactive Protein and Tumor Necrosis Factor-Alpha in Differentiating between Ventilator-Associated Pneumonia and Systemic Inflammatory Response Syndrome without Infectious Etiology

**Published:** 2016

**Authors:** Ebrahim Salehifar, Shima Tavakolian Arjmand, Masoud Aliyali, Siavash Abedi, Ali Sharifpour, Abbas Alipour, Shahram Ala, Gohar Eslami, Farzad Bozorgi, Mohammad Reza Mahdavi, Keith R. Walley

**Affiliations:** 1 Department of Pharmacotherapy, Faculty of Pharmacy, Mazandaran University of Medical Sciences, Sari, Iran; 2 Department of Pharmacotherapy, Faculty of Pharmacy, Mazandaran University of Medical Sciences, Sari, Iran; 3 Department of Internal Medicine, Faculty of Medicine, Mazandaran University of Medical Sciences, Sari, Iran; 4 Department of Epidemiology and Community Medicine, Faculty of Medicine, Mazandaran University of Medical Sciences, Sari, Iran; 5 Department of Emergency Medicine, Faculty of Medicine, Mazandaran University of Medical Sciences, Sari, Iran; 6 Department of Laboratory Tests, Mazandaran University of Medical Sciences, Sari, Iran; 7 Centre for Heart Lung Innovation, University of British Columbia, Vancouver, BC, Canada

**Keywords:** Biomarker, Infection, Inflammation, Systemic inflammatory response syndrome, Ventilator-Associated Pneumonia

## Abstract

**Background:**

Differential diagnosis of systemic inflammatory response syndrome (SIRS) with or without infectious cause is critically important in terms of initiating antimicrobial agents in case of infectious etiology such as ventilator-associated pneumonia (VAP). The aim of this study was to determine the diagnostic and prognostic roles of C-reactive protein (CRP) and tumor necrosis factor-alpha (TNF-α) in differentiating between ventilator-associated pneumonia and SIRS without infectious etiology.

**Materials and Methods:**

In this prospective observational study, 91 adult intensive care unit (ICU) patients were enrolled. According to established diagnostic criteria, they were classified into three groups of “non-SIRS non-VAP”, “SIRS non-VAP” and “SIRS-VAP”. Serum CRP and TNF-α were measured on days 1, 3 and 7 of the study and compared using repeated measures ANOVA.

**Results:**

With respect to diagnosis, there was no significant difference in the values of these biomarkers between groups (P>0.05). There was no statistically significant “time trend” for C-reactive protein and TNF-α (P>0.05). Considering both group effect and Time effect, the changes were not significantly different for CRP (P= 0.86) and TNF-α (P=0.69). In contrast, the clinical score and the clinical pulmonary infection score (CPIS) ≥ 6, had 100% specificity for diagnosing VAP. With respect to prognosis, only an unchanged or decreasing TNF-α from day 1 to day 3 was marginally associated with 28-day survival. However, day 1 and day 3 acute physiology and chronic health evaluation II (APACHE II) scores were highly associated with 28-day survival.

**Conclusion:**

Unlike clinical scoring system including CPIS and APACHE II, TNF-α and CRP levels were not useful as diagnostic or prognostic biomarkers for differentiating between SIRS with VAP etiology and SIRS without infectious etiology.

## INTRODUCTION

Biomarker measurement in critically ill patients has received increasing attention (1, 2). Biomarkers can aid in diagnosis or prognosis (1). For example, in a clinical ICU setting the measurement of CRP is now included in the surviving sepsis campaign international guidelines as a component of the diagnosis of sepsis (3). Frequent biomarker measurements in critically ill patients have been reported to be prognostic of outcome (1). For example, elevated CRP levels have been reported to be prognostic of increased mortality in critically ill septic patients (4, 5). Too frequently, these studies omit the comparison of the measured value of biomarker to the value of existing clinical scoring systems. Thus, it is often unclear whether biomarker measurement adds to clinical management and, in particular, if biomarker measurement is most helpful in establishing a diagnosis or by improving prognostic estimates.

To address these issues, we chose to study a common yet challenging problem in the ICU – the diagnosis of VAP. VAP is a very common cause of morbidity and mortality in ICU patients (2, 6, 7). The clinical diagnosis of VAP is usually based on systemic signs of infection, new or expanding pulmonary infiltrates seen on chest roentgenogram and bacteriologic evidence of pulmonary parenchymal infection (6). Although, microbiologic diagnosis of VAP is crucial for specific diagnosis and management, it takes a substantial period of time to obtain culture results. Thus, VAP is an important ICU problem that would greatly benefit from enhanced diagnostic capacity provided by rapid biomarker measurements (8–10).

CRP measurement helps the diagnosis of infection (11). However, specificity for infection has been raised as a limitation (12). CRP is an acute-phase protein produced by the liver, the levels of which rise in response to inflammation. CRP concentrations below ∼10 mg/L are considered normal. CRP increases slightly with age, pregnancy, various kinds of mild inflammation and viral infections (10–40 mg/L). Higher levels are observed in severe bacterial infections. In contrast to CRP, TNF-α is an early indicator of inflammation (13) arising from both infectious and non-infectious causes. TNF-α plays an important role in pathophysiology of many inflammatory disorders, whether due to infection or due to non-infectious causes (12, 14–16).

We therefore interrogated the diagnostic and prognostic value of both CRP and TNF-α in enhancing the clinical distinction between 1) critically ill patients with and without a clinically defined SIRS, and 2) with and without infection (17–19). To address these issues, we chose to follow a prospective cohort of critically ill patients for the development of VAP. From a diagnostic perspective, we reasoned that TNF-α should distinguish between patients with SIRS and without SIRS (non-SIRS) while CRP should perform best at distinguishing infection (VAP) from no infection (non-VAP). We compared the diagnostic ability of these two prototype biomarkers to simple clinical scores. Finally, we separately tested the prognostic value of these measurements, again compared to prognosis from simple clinical scores. These prospective and carefully timed measurements raise doubt as to whether current biomarkers add substantially to current clinical practice in VAP diagnosis and prognosis.

## MATERIALS AND METHODS

This study was carried out from March 2012 to February 2013 in the ICUs of a university hospital, in Sari, Iran. There are three intensive care units in the hospital including medical (12 beds), surgical (eight beds) and gynecological (six beds) ICUs. The study was approved by the Review Board and Ethics Committee of Research Deputy of Mazandaran University of Medical Sciences (No: 91-103). All patients or their first relatives (e.g., for unconscious patients) were given information about the aims and methods of the study and informed consent was signed by them before enrolling the study.

All patients with an ICU stay of greater than 72 hours were eligible for inclusion. One hundred and twenty-seven ICU patients were screened for potential inclusion. The exclusion criteria were 1) discharged or death within 72 hours of ICU admission (n=16), 2) obstetric patients admitted to the ICU following delivery (n=5), 3) diagnosis of pneumonia less than 48 hours after intubation (these patients were considered to have community acquired pneumonia, not VAP) (n=7), 4) patients receiving chronic corticosteroid treatment (n=6), and 5) human immunodeficiency virus positive patients (n=2). With these exclusions, 91 ICU patients were enrolled for further study. Patients were then followed daily for the duration of their ICU stay for the occurrence of VAP.

SIRS was defined using American College of Chest Physicians (ACCP) recommendations criteria. That is, patients were considered to have SIRS if they met two or more of the following conditions: 1) temperature ≥38 or ≤36°C, 2) heart rate ≥90 beats/min, 3) respiratory rate ≥20 breaths/min or PaCO_2_ ≤32 mmHg, and 4) white blood cell count ≥12,000 or ≤4,000/mL, or presence of more than 10% immature neutrophils (20, 21). VAP was defined by the presence of mechanical ventilation, SIRS, a CPIS of greater than or equal to 6 and, subsequently, a positive endotracheal aspirate culture (6). Endotracheal aspirate cultures were only performed in patients who had a CPIS≥6 (22).

Patients were then categorized into the following three groups based on the absence or presence of systemic inflammatory response syndrome and the absence or presence of VAP as follows. a) The non-SIRS-non-VAP group did not fulfill the definition of SIRS and did not fulfill the definition of VAP (n = 22). b) The SIRS-non-VAP group included patients who fulfilled the definition of SIRS but did not fulfill the definition of VAP (n=39). c) The SIRS-VAP group fulfilled the definition of SIRS and fulfilled the definition of VAP (n=30).

Demographic data were recorded at the time of ICU admission. For non-VAP patients (non-SIRS-non-VAP and SIRS-non-VAP) the day of ICU admission was defined as the first study day. For patients who subsequently developed VAP, the day of VAP development was defined as the first study day. Data were collected in the first, third and seventh study days. These data comprised the following items: Temperature, heart rate, respiratory rate, blood pressure, laboratory analysis including white blood cell count (WBC), percentage of neutrophils and band forms, serum creatinine, arterial blood gas analysis, and plasma CRP and TNF-α concentrations. In addition, CPIS and APACHE II scores were determined (23).

### CRP and TNF-α measurements

Blood samples were drawn on the first, third and seventh study days for CRP and TNF-α measurements. Blood samples were collected in glass tubes and placed in ice containers. Samples were processed within two hours by centrifuging at 1,600 g for 15 minutes. Plasma supernatant was rapidly frozen and preserved at −70°C until final analysis. CRP concentration was measured using a particle enhanced turbidimetris assay (Roche, Germany) and TNF-α concentration was measured using an enzyme-linked immune sorbent assay (ELISA. eBioscience, Austria).

### Statistical analysis

We used the Shapiro-Wilk test to determine whether data were normally distributed. Descriptive baseline characteristics for comparison of the three groups (non-SIRS-non-VAP, SIRS-non-VAP, SIRS-VAP) were tabulated as mean ± standard deviation (SD) or as percentages. To compare the three groups, chi-square test was used for categorical data and ANOVA or Kruskal-Wallis test was used for continuous data. Using a general linear model, CRP and TNF-α concentrations were compared among the three groups using two-way repeated measures ANOVA. Time of evaluation (days 1, 3 and 7) was the repeated factor and patient group (non-SIRS-non-VAP, SIRS-non-VAP, SIRS-VAP) was the group factor. We used Mauchly’s sphericity to test the compound symmetry assumption. Additionally, we used a logistic regression analysis to determine the association between survival status on day 28 and biomarker concentrations. Changes in biomarker concentrations were divided into dichotomous variables (unchanged/decreased versus increased at two times after ICU admission), and were entered into univariate and multivariate analyses. We entered the variables with biological importance and variables with P < 0.20 in univariate analysis to multivariable logistic regression analysis. In multivariate logistic regression analysis, a P value of 0.05 or less was considered statistically significant. Data were analyzed using SPSS statistics version 16 and Stata version 10.

## RESULTS

A total of 91 patients (69 males and 22 females with a mean age of 51.9 ± 22.4 years) who fulfilled the inclusion/exclusion criteria were studied in this prospective observational clinical trial. Twenty-two patients were categorized as non-SIRS-non-VAP, 39 as SIRS-non-VAP, and 30 as SIRS-VAP. Demographic and clinical characteristics are shown in Table 1.

**Table 1.** Baseline demographics and clinical characteristics of patients

**Variables**	**Non-SIRS-non-VAP n=22**	**SIRS-non-VAP n=39**	**SIRS-VAP n=30**	**P value**
**Age, years**	52.8±20.4	49.9±24.7	53.0±22.1	0.82
**Sex (M/F)**	16/6	29/10	24/6	0.8
**Length of stay in hospital prior to admission to ICU; days**	3.6±6.4	2.8±5.9	1.4±4.1	0.34
**Use of antibiotics prior to admission to ICU; n (%)**	13 (59.1)	30 (76.9)	25 (86.3)	0.08
**APACHE II score**	15.4±8.0	17.3±7.0	16.4±6.8	0.58
**Mechanically ventilated; n (%)**	8 (36%)	25 (64%)	30 (100)	< 0.001
**Days on mechanical ventilation during a 28-day stay in ICU**	3.5±7.3	6.5±7.9	19.5±9.8	< 0.001

APACHE II: Acute physiology and chronic health evaluation II; SIRS: Systemic inflammatory response syndrome; VAP: Ventilator-associated pneumonia

### Biomarkers as diagnostic factors

**Figure 1.**
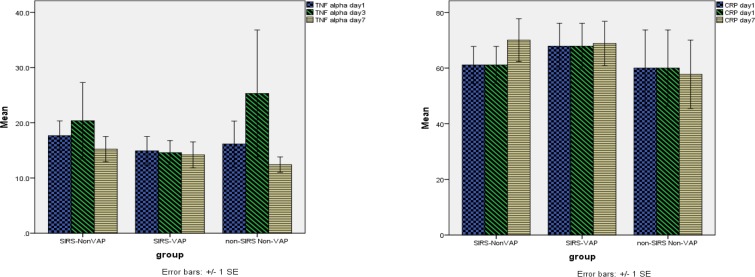
C-reactive protein and Tumor necrosis factor-α concentrations at study days 1, 3, and 7 in NonSIRS-NonVAP, SIRS-NonVAP, and SIRS-VAP patients.

There were no significant differences in the values of CRP and TNF-α between groups (between-subject differences or group effect) (Figure 1). In addition, there was no statistically significant time trend (within-subject differences or time effect) for CRP and TNF-α (Figure 1). Considering both time effect and group effect, the changes were not significantly different for CRP (P= 0.86) and TNF-α (P=0.69). Thus, TNF-α was not effective in diagnosing SIRS, and CRP was not effective in diagnosing VAP in patients who had SIRS.

**Figure 2.**
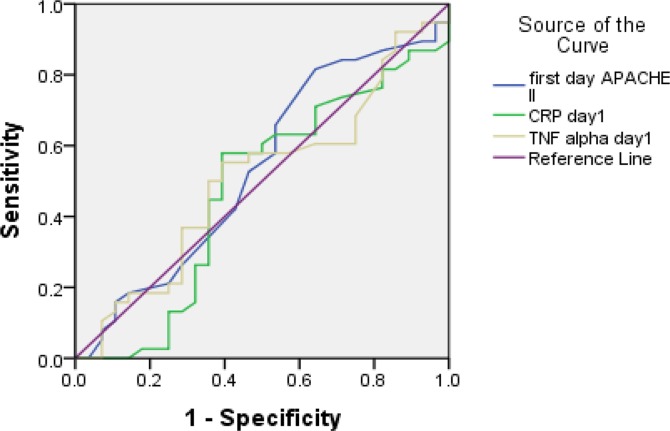
Receiver operating characteristic curve for sensitivity and specificity of C-reactive protein and tumor necrosis factor-alpha at days 1, 3, and 7 of diagnosis for discriminating SIRS-nonVAP from SIRS-VAP

We used ROC analysis to calculate the sensitivity and specificity of CRP to differentiate SIRS-non-VAP from SIRS-VAP and similar analysis for TNF-α. Table 2 and Figure 2 show the optimal cut-offs and corresponding sensitivities, specificities and area under the curve for CRP and TNF-α. At days 1, 3 and 7, TNF-α had sensitivity values >0.7, but the CRP at all times reached sensitivity <0.7. Considering the specificity, just CRP level at day 1 showed specificity >0.7. The area under the curve of both CRP and TNF-α on each given days was not significantly different from 0.5.

**Table 2:** Area under the curve as well as sensitivity and specificity for the chosen cut-off levels of C-reactive protein and Tumor necrosis factor-alpha at 1, 3 and 7 days after ICU admission for the end point condition (SIRS-non-VAP and SIRS-VAP)

	Cut-off	Sensitivity	Specificity	AUC	P value
CRP day 1	87.5	33.3	77.3	0.521	0.81
CRP day 3	63.5	45.8	54.5	0.483	0.84
CRP day 7	73.5	45.8	63.6	0.473	0.75
TNF-α day 1	8.4	70.8	36.4	0.456	0.61
TNF-α day 3	8.25	79.2	31.8	0.451	0.57
TNF-α day 7	7.5	87.5	31.8	0.491	0.91
APACHE II day 1	15	0.65	0.52	0.594	0.29
APACHE II day 3	16.5	0.61	0.57	0.521	0.81
APACHE II day 7	8	0.83	0.24	0.432	0.44

APACHE II: Acute physiology and chronic health evaluation II; AUC: Area under the curve; CRP: C-reactive protein; ICU: Intensive care unit; SIRS: Systemic inflammatory response syndrome; TNF-α: Tumor necrosis factor- alpha; VAP: Ventilator-associated pneumonia

In contrast to these biomarkers, we found that every patient who had CPIS ≥ 6 had positive endotracheal aspirate cultures and, hence, VAP. Thus, in this setting CPIS ≥ 6 had 100% specificity.

Systemic Inflammatory Response Syndrome (SIRS); Ventilator-Associated Pneumonia (VAP). There was no significant difference in the values of CRP and TNF-α between groups (between-subject differences or group effect) and there was no statistically significant time trend (within-subject differences or time effect) for CRP and TNF-α. Considering both time effect and group effect, the changes were not significantly different for CRP (P= 0.86) and TNF-α (P=0.69).

### Biomarkers as prognostic factors

Two, eight and 17 patients died by days three, seven and 28 after ICU admission, respectively. We tested early biomarker levels (day 1 and day 3) for prognostic value of mortality, reasoning that many deaths had occurred by day 7 and day 7 was late in the typical course of VAP. Biomarker levels (median and range) in survivors and non-survivors (28 days after study inclusion) on day one and day three are shown in Table 3. While CRP and TNF-α were not predictive of mortality, APACHE II scores were significantly different between survivors and non-survivors on day one (P=0.008) and day three (P=0.005).

**Table 3.** Biomarker levels (median and percentile range 25–75%) in survivors and non-survivors

**Biomarkers**	**Non-survivors (n=17)**	**Survivors (n=74)**	**P value**
**CRP day 1**	40 (23–80)	60.5 (32–84.8)	0.48
**CRP day 3**	43 (28.3–84)	46 (29.5–76)	1
**TNF-α day 1**	12 (8–22.2)	9.2 (7.8–18.4)	0.41
**TNF-α day 3**	9.1 (7.7–14.7)	10.2 (8–18)	0.35
**APACHE II day 1**	21 (16.3–25)	15.5 (11.8–20)	0.008
**APACHE II day 3**	18 (16–22)	15 (10–18)	0.005

APACHE II: Acute physiology and chronic health evaluation II; CRP: C-reactive protein; TNF-α: Tumor necrosis factor- alpha; VAP: Ventilator-associated pneumonia

No change or reduction in TNF-α occurred in 39 of 74 (59.1%) survivors but only in 4 of 17 (25.2%) non-survivors (P=0.014). No change or reduction in CRP from day 1 to day 3 occurred in 43 of 74 (64.2%) survivors and in 7 of 17 (43.8%) non-survivors (P=0.13). A multivariable logistic regression model for survival status revealed that only no change or reduction in TNF-α from day 1 to day 3 (OR= 10.2, 95% confidence interval 25 to 100, P = 0.044) and APACHE II (OR= 6.58, 1.15 to 37.0, P = 0.034) remained significant.

## DISCUSSION

Diagnosis of sepsis and, hence, the need to initiate antibiotic therapy is crucial for good clinical outcomes. New sepsis guidelines propose monitoring of CRP and serum cytokines to aid in the diagnosis of sepsis in critically-ill patients in the ICU. To test these proposals directly we chose VAP as an important example in the ICU and measured CRP and TNF-α concentrations over the first seven days. We compared these measurements to clinical scoring systems to determine whether they contributed to establishing a diagnosis or improving prognostic estimates.

With regard to diagnosis we found that CPIS ≥ 6 had 100% specificity for diagnosing VAP. However, CRP and TNF-α during the first week of ICU stay did not distinguish between critically-ill patients without SIRS or VAP (non-SIRS-non-VAP), patients with SIRS but no VAP (SIRS-non-VAP), and patients who had SIRS and VAP (SIRS-VAP). Following changes in these biomarkers over time did not show diagnostic ability either.

With regard to prognosis we found that clinical scoring using the APACHE II score was a highly significant predictor of 28-day mortality. In contrast, neither CRP nor TNF-α were predictive of 28-day mortality. In further analysis, we found that no change or reduction in concentration of TNF-α from day 1 to day 3 was more common in survivors than non-survivors. Hillas et al, also demonstrated that higher level of CRP at day 7 in VAP patients was associated with development of septic shock, although it could not predict VAP survival (24).

The sensitivity of CRP on day 1 to discriminate “SIRS-non-VAP” from “SIRS-VAP” was very low (e.g., 33.3%). In most previous studies, CRP levels have been referred to be an indicator of morbidity and mortality rather than a diagnostic test (10, 11, 17, 25, 26). Póvoa et al. found that in community acquired sepsis patients admitted to the ICU, the survivors had a lower CRP level on days three to five of stay in the ICU compared to non-survivors (11). The pattern of CRP changes could predict the postoperative complications and higher one-year mortality in patients undergoing esophagectomy (10). CRP levels more than 10 mg/dL were associated with 6.6 times higher mortality in respiratory ICU patients (25). Considering the prognostic utility of CRP, it was used successfully for assessing response to antibiotics (8), risk stratification of cardiovascular disease (17) and for predicting acute brain dysfunction in critically-ill patients (26).

In cardiac surgery patients, serum CRP was not a diagnostic marker for VAP although procalcitonin was (27). Similar results of preference of procalcitonin over CRP was reported in discriminating SIRS and sepsis (18) and also as a marker for detection of early VAP (9) or VAP in patients with a successful cardiopulmonary resuscitation (28). The assay of CRP in bronchoalveolar lavage fluid was also not helpful in the diagnosis of VAP (29). Decrease in CRP, as decrease in PCT, Sequential Organ Failure Assessment and APACHE II was associated with the prediction of survival of VAP patients (30). APACHE II is an established and feasible outcome predictor tool in critically-ill patients including septic patients who are at high risk of death and who are more likely to benefit from intervention (31, 32). We found that APACHE II is a better predictor of survival compared to CRP and TNF-α.

In our study, TNF-α, similar to CRP, could not differentiate between “SIRS-VAP” and “SIRS-non-VAP”, although higher TNF-α on day three was associated with a higher mortality. The sensitivity of TNF-α at all three time points was more than 70%, but the area under the curves of sensitivity and specificity was not significantly different from 0.5. In most studies, the trend of inflammatory mediators was compared between SIRS and sepsis/septic shock (19, 33). A higher level of TNF-α was observed in sepsis patients compared to SIRS or control groups. Also, higher TNF-α level was associated with higher occurrence of disseminated intravascular coagulation and mortality. In contrast, it was reported that TNF-α dynamics were not associated with risk estimation of mortality in SIRS of infectious origin (19). In our study, we found a prognostic role for TNF-α at D3, as higher level was inversely associated with survival, irrespective of the primary cause of inflammation.

Recently, simultaneous use of several inflammatory markers including cell-surface (e.g., triggering receptor expressed by myeloid cells-1, CD11b and CD62L) and soluble markers (IL-1beta, IL-6, IL-8, sTREM-1, Procalcitonin) has been used successfully to discriminate between VAP and non-VAP cases (34). One limitation of our study was that we did not measure some other biomarkers such as IL-6 and IL-8 in our study.

The overall conclusion of the study is that TNF-α and CRP levels were not capable of differentiating “SIRS-non-VAP” from “SIRS-VAP” patients. Instead, a readily available diagnostic scoring system, CPIS ≥ 6, had 100% specificity for diagnosing VAP. Similarly, TNF-α and CRP levels had little prognostic ability while a standard clinical severity of illness scoring system, APACHE II, was significantly prognostic. These prospective and carefully timed measurements raise doubt as to whether current biomarkers add substantially to current clinical practice in VAP diagnosis and prognosis.
